# Fusion of metabolomics and proteomics data for biomarkers discovery: case study on the experimental autoimmune encephalomyelitis

**DOI:** 10.1186/1471-2105-12-254

**Published:** 2011-06-22

**Authors:** Lionel Blanchet, Agnieszka Smolinska, Amos Attali, Marcel P Stoop, Kirsten AM Ampt, Hans van Aken, Ernst Suidgeest, Tinka Tuinstra, Sybren S Wijmenga, Theo Luider, Lutgarde MC Buydens

**Affiliations:** 1 Radboud University Nijmegen, Institute for Molecules and Materials, Heyendaalseweg 135, 6524 NP Nijmegen, The Netherlands; 2 Abbott Healthcare Pharmaceuticals Nederland B.V., C.J. van Houtenlaan 36, 1381 CP Weesp, The Netherlands; 3 Department of Neurology, Erasmus University Medical Center Rotterdam, Dr. Molewaterplein 50, 3015 GE Rotterdam, The Netherlands

## Abstract

**Background:**

Analysis of Cerebrospinal Fluid (CSF) samples holds great promise to diagnose neurological pathologies and gain insight into the molecular background of these pathologies. Proteomics and metabolomics methods provide invaluable information on the biomolecular content of CSF and thereby on the possible status of the central nervous system, including neurological pathologies. The combined information provides a more complete description of CSF content. Extracting the full combined information requires a combined analysis of different datasets i.e. fusion of the data.

**Results:**

A novel fusion method is presented and applied to proteomics and metabolomics data from a pre-clinical model of multiple sclerosis: an Experimental Autoimmune Encephalomyelitis (EAE) model in rats. The method follows a mid-level fusion architecture. The relevant information is extracted per platform using extended canonical variates analysis. The results are subsequently merged in order to be analyzed jointly. We find that the combined proteome and metabolome data allow for the efficient and reliable discrimination between healthy, peripherally inflamed rats, and rats at the onset of the EAE. The predicted accuracy reaches 89% on a test set. The important variables (metabolites and proteins) in this model are known to be linked to EAE and/or multiple sclerosis.

**Conclusions:**

Fusion of proteomics and metabolomics data is possible. The main issues of high-dimensionality and missing values are overcome. The outcome leads to higher accuracy in prediction and more exhaustive description of the disease profile. The biological interpretation of the involved variables validates our fusion approach.

## Background

The omics fields hold huge promises in the investigation of various diseases. Multiple examples are available in the literature showing the potential of proteomics [1,2] and metabolomics [3]. Both domains carry valuable information about biological pathways involved in human diseases [4,5]. Proteomics aims to provide a comprehensive identification and quantification of proteins present in tissues or biofluids. This information allows one to find networks of cellular mechanisms and to get more insight into molecular disease processes [6]. Metabolomics aims to determine the small molecule fingerprints of cellular processes. Similarly to proteomics, metabolomics relies on the detection and quantification of small biomolecules, here metabolites. The proteome and metabolome separately and in combination represent an instant picture of a biological system [7].

The proteome and metabolome are not disjoint. Protein levels obviously influence the metabolic profile, *e.g*. through enzymatic reactions. On the other hand, the metabolites concentrations may affect the expression of proteins [8]. Therefore, the combination of this information in a systems biology approach is expected to provide a more comprehensive understanding of biological entities [9]. Various studies have been proposed to analyse jointly different metabolomics or proteomics data in order to improve the overall understanding of the system [10,11]. Our aim is here to propose a new statistical approach. The objective of this method is to perform data fusion of multiple types of measurements related to the same biological system. We chose a proteomics and metabolomics platform that measured the very same CSF samples. This biofluid is in close interaction with the Central Nervous System (CNS). Therefore, CSF can be expected to reflect the status of the CNS. This assumption has been used to study neurological diseases such as Multiple Sclerosis (MScl) [12]. MScl is an autoimmune disease and the most common cause of neurological disability in young adults [13]. It manifests itself as an acute, focal, inflammatory demyelination and axonal loss with limited remyelination. The disease culminates in chronic multifocal sclerotic plaques. The associated symptoms are various and follow an unpredictable course. The CSF samples are relatively accessible. However, collecting samples from large cohorts of MScl patients and healthy volunteer is still a complex and time consuming task. Moreover, the analysis of such clinical samples often encounters challenges related to the variation linked to genetic and environmental background.

In the frame of biomarker discovery, it is therefore more straightforward and productive to initiate an investigation from an animal model of the disease. The Experimental Autoimmune Encephalomyelitis (EAE) model is one of the most intensively studied model of autoimmune disease [14]. EAE, like MScl is an inflammatory disorder of CNS. It is mainly used to look at the neuroinflammatory mechanism, which is one of the key hallmarks of MScl. This animal model has been reproduced in many species, including rats [15]. Depending on the species and antigen used, the resulting EAE presents a specific disease course. The acute version of this model, as used in this study, is induced by the injection of Complete Freund Adjuvant (CFA) and Myelin Basic Protein (MBP). The initial symptom, paralysis of the tip of the rat's tail, appears after approximately 10 days. This ultimately culminates in paralysis extended up to the limbs. The 10th day can be considered as the onset of disease. Our main interest in this study is to define the combined proteomic and metabolomic biomarker pattern corresponding to this onset. An experimental design has been constructed to characterize the disease at this time point. The aim is to establish whether these combined biomarkers and/or biomarker profiles can act as predictive indicators of the onset of disease.

The main objective from a data analysis point of view is to extract from both proteome and metabolome all the relevant information allowing a comprehensive definition of the disease profile. It is also of the utmost importance to extract information from both platforms. Indeed a shared pattern would provide insights on interactions between proteins and metabolites. This information could greatly enhance our comprehension of the disease process. For this purpose, we propose a so-called mid level fusion architecture [16] based on two layers. The first layer aims to extract all the information allowing for discrimination between control and disease samples per platform. This step is hampered by the usual "curse of dimensionality", as it will be shown in the next section. The second layer is designed to fuse the information of each platform. At this point, a new problem arises related to missing measurements from platform to platform. To solve these practical problems, we propose a solution based on two methods: extended Canonical Variates Analysis (eCVA) [17] and a modified version of Principal Component Analysis (PCA) [18]. These two methods and our data analysis strategy are described in the methods section. The fusion method is then applied to the analysis of metabolomics and proteomics data of the EAE experiment. The outcome of this approach allows for a better description of the data of the joint platforms than the one achieved by using the data from the two platforms separately. The variables driving the model are then explored in term of biological meaning. This allows confirming the validity of our approach. In addition, it is possible to visualize the EAE biomarker profile on the systems biology level.

## Methods

### Material

#### Induction of Acute EAE in Lewis rat

Male Lewis rats (Harlan Laboratories B.V., the Netherlands) kept under normal housing conditions with water and food *ad libitum*, weighing between 175 and 225 grams at the start of the experiment, were inoculated on day 0 as previously described [19]. Briefly, a 100 μL saline based emulsion containing 50 μL Complete Freund Adjuvant H37 RA (CFA, Difco Laboratories, Detroit, MI), 500 μg Mycobacterium tuberculosis type H37RA (Difco) and 20 μg guinea pig Myelin Basic Protein (MBP) was injected subcutaneously in the pad of the left hind paw of Isoflurane anaesthetized animals. Next to these MBP challenged rats, referred to as the EAE group, two control groups were included: a group of animals receiving the same emulsion without MBP (CFA group) and a healthy group undergoing anesthesia only (Healthy group). Each group consisted of 15 animals. The animals present in one given cage were all belonging to the same group. The animals were sacrificed to collect CSF on day 10 (day of onset in the EAE group). The design is summarized in Table 1.

**Table 1:** Experimental Design of EAE model.

Treatment day 0	Groups number	Number of animals	Effects
Anesthesia only	1	15	Healthy

CFA	2	15	Peripheral Inflammation

CFA+MBP	3	15	Neuroinflammation (EAE) + peripheral inflammation

Animals were housed per 3 and cages were randomized across treatments and disease duration. Disease symptoms and weights of all animals were recorded daily. The animal experiments described were approved by the local Institutional Animal Care and Use Committee.

#### CSF sampling

On day 10, animals were euthanized with CO_2_/O_2_. Terminal CSF samples were obtained by direct insertion of an insulin syringe needle (Myjector, 29 G × 1/2") via the arachnoid membrane into the Cisterna Magna. For this purpose a skin incision was made followed by a horizontal incision in *the musculus trapezius pars descendens *to reveal the arachnoid membrane. A volume of maximum 60 μL was collected per animal. Each sample was centrifuged within 20 min after sampling, for 10 min at 2000 g at 4°C. After centrifugation the supernatants were stored at -80°C for further analysis. Previous experiments have shown that collecting up to 60 μL using this technique and conditions provided hemoglobin-free CSF samples measured by ESI-Orbitrap. As an additional check fresh samples, supernatant and pellet size were visually scored for hemolysis and samples were discarded if positive.

From the set of 45 samples, 3 of them (from different groups) were contaminated with blood and were excluded from measurements.

### Measurements

#### MS-Orbitrap: samples preparation and data acquisition

10 μL of rat CSF sample was put into a 96-microtiter-well plate (Nunc low binding, VWR, The Netherlands), and 20 μL of 0.2% Rapigest (Waters, Milford, MA) in 50 mM ammonium bicarbonate buffer was added to each well. After a 30 min incubation period with 1,4-dithiothreitol (60°C) and, subsequently, iodoacetamide (37°C), 4 μL of 0.1 μg/μL gold-grade trypsin (Promega, Madison, WI)/3 mM Tris-HCl (pH 8.0) was added to each sample. The samples were incubated overnight at 37°C. To adjust the pH of the digest to pH < 2, a high concentration of trifluoroacetic acid was added to the mixture prior to a final incubation step at 37°C for a duration of 45 minutes to stop the digestion reaction.

Mass spectrometry measurements were carried out on a Ultimate 3000 nano LC system (Dionex, Germering, Germany) online coupled to a hybrid linear ion trap/Orbitrap MS (LTQ Orbitrap XL; Thermo Fisher Scientific, Germany). 5 μL digest were loaded onto a C18 trap column (C18 PepMap, 300 μm ID × 5 mm, 5 μm particle size, 100 Å pore size; Dionex, The Netherlands) and desalted for 10 minutes using a flow rate of 20 μL/min 0.1% TFA. Then the trap column was switched online with the analytical column (PepMap C18, 75 μm ID × 150 mm, 3 μm particle and 100 Å pore size; Dionex, The Netherlands) and peptides were eluted with the following binary gradient: 0%-25% solvent B for 120 min and 25%-50% solvent B for a further 60 minutes, where solvent A consist of 2% acetonitrile and 0.1% formic in water and solvent B consists of 80% acetonitrile and 0.08% formic acid in water. The column flow rate was set to 300 nL/min. For MS detection a data dependent acquisition method was used: high resolution survey scan from 400-1800 Th. was performed in the Orbitrap (value of target of automatic gain control AGC 10^6^, resolution 30,000 at 400 m/z; lock mass was set to 445.120025 u (protonated (Si(CH_3_)_2_O)_6_) [20]). Based on this survey scan the 5 most intensive ions were consecutively isolated (AGC target set to 10^4 ^ions) and fragmented by collision-activated dissociation (CAD) applying 35% normalized collision energy in the linear ion trap. After precursors were selected for MS/MS, they were excluded for further MS/MS spectra for 3 minutes.

Following a standardized noise reduction procedure, the Orbitrap raw files were preprocessed using the Progenesis LC-MS software package (version 2.5, Nonlinear Dynamics, Newcastle-upon-Tyne, United Kingdom). In this software package all peaks in the raw files are aligned according to their retention time by a graphical detection algorithm. This algorithm detects the peptide peaks in a gel-view representation of the mass spectrometry data and matches corresponding peaks (termed as features in this software package) between samples. Strict criteria for alignment acceptance were employed (at least 200 corresponding peaks per sample for the sample to be included in subsequent analysis steps). Following this, peptides were identified and assigned to proteins by exporting features, for which MS/MS spectra were recorded, using the Bioworks software package (version 3.2; Thermo Fisher Scientific, Germany; peak picking by Extract_msn, default settings). The resulting. mgf file was submitted to Mascot (version 2, Matrix Science, London, United Kingdom) for identification to interrogate the UniProt-database (version 57.0, taxonomy (*Rattus norvegicus*): 7114 sequences). Only ions with charge states between +2 and +7 were considered and only proteins with at least two unique peptides (Mascot sore > 25) assigned to them were accepted as true identifications. Carbamidomethylation of cysteine was set as fixed and oxidation of methionine as variable modification allowing a maximum of 2 missed cleavages. Mass tolerance for precursor ions was set to 10 ppm and for fragment ions at 0.5 Da. The cut-off for mass differences between the measured and the theoretical mass of the identified peptides was set at 2 ppm. The Mascot search results were imported back into the Progenesis software to link the identified peptides to the detected abundances of these peptides. The data were exported subsequently in Excel format. In this exported excel file an abundance (area-under-the-curve) is listed for all features (and consequently all identified peptides and proteins) in all individual samples. The abundance is used instead of the peak intensity to account for tailing of the peptide peaks during the LC-separation. The differences in abundance are subsequently used for analysis of the differences between the groups for all identified peptides and proteins.

#### NMR: samples preparation and data acquisition

10 μL of rat CSF were thawed at room temperature and 240 μL D_2_O were added to biofluid in order to obtain the sufficient amount of sample for NMR measurement. We used TSP-d4 (99 at.%D) as an internal standard for chemical shift reference (δ 0.00 ppm) and metabolite quantification. For the latter, 25 μL of 8.8 mM TSP-d4 stock solution in D_2_O was added to 250 μL of rat CSF to a final concentration of 0.8 mM TSP. The TSP-d4 stock was prepared by weighing in dry TSP-d4. The pH of the CSF was adjusted to around 7 (7.0 - 7.1) by adding phosphate buffer (9.7 μL 1 M, to a final concentration of 35 mM). The final CSF NMR sample (284.7 μL) was then transferred to a SHIGEMI microcell NMR tube for NMR measurements.

The 1D ^1^H NMR spectra of rat CSF samples were acquired on an 800 MHz Inova (Varian) system equipped with a 5 mm triple-resonance, Z-gradient HCN cold-probe. Suppression of water was achieved by using WATERGATE (delay: 85 μs). For each 1D ^1^H NMR spectrum 512 scans of 18 K data points were accumulated with a spectral width of 9000 Hz. The acquisition time for each scan was 2 s. Between scans, a 8 s relaxation delay was employed. Prior to spectral analysis, all acquired Free Induction Decays (FIDs) were zero-filled to 32 K data points, multiplied with a 0.3 Hz line broadening function, Fourier transformed, manually phase and the TSP internal reference peak was set at 0 ppm - by using ACD/SpecManager software. The set of 42 rat CSF spectra were acquired and preprocessed as described above. However due to high line broadening of internal standard (TSP) two spectra were not included in spectral analysis. In total 40 spectra were subsequently transferred to Matlab, version 7.6 (R2008b) (Mathworks, Natick, MA) for further analysis.

The NMR spectral data is then preprocessed, which typically involves baseline correction, alignment, binning, normalization and scaling. Baseline correction of NMR spectra was performed by applying Asymmetric Least Square method [21]. Fluctuation in experimental conditions like sample temperature, pH and ionic strength can lead to chemical shift variations, therefore NMR spectra were aligned by using improved parametric time warping [22]. A further problem is the high dimensionality of the data (circa 10000 variables). It is common to apply binning to this kind of data which reduces the number of variables. To perform proper spectral bucketing we used adaptive intelligent binning [23]. This binning procedure, more complex than the classical binning, ensures that peaks are not divided over multiple bins. Moreover it allows excluding regions without signals from the analysis. The chemical shift range δ 0.75 - 4.15 was used for binning procedure because it contained most relevant information. This region was selected based on visual inspection of the spectra because it contains signals with high signal to noise ratio. Next, spectral resonances corresponding to one identified metabolite were summed and regrouped in one bin. This procedure was applied to the resonances were no overlapping was present and it led to 153 bins in total. The identification of the metabolites was performed using Chenomx (Edmonton, Canada). For the purpose of making spectra comparable as the final step of the preprocessing approach integral normalization was applied to the binned data.

The analysis proposed in this work is initiated by an exploratory analysis. The objective here is to assess the quality of the two data sets and detect outliers. The fusion itself is performed afterwards. Both steps are detailed below, respectively.

#### Exploratory method

The different data sets have been analyzed using exploratory methods in order to detect outliers. This also provides some insights on the information content of the data sets and eventually their ability to provide relevant information related to the separation of the groups. For this purpose, Principal Component Analysis (PCA) [24] and robust PCA [25] have been used. The outliers detection has been based on cut off values on both orthogonal and Mahalanobis distances, as proposed in [26].

#### Mid-level fusion

**Figure 1:**
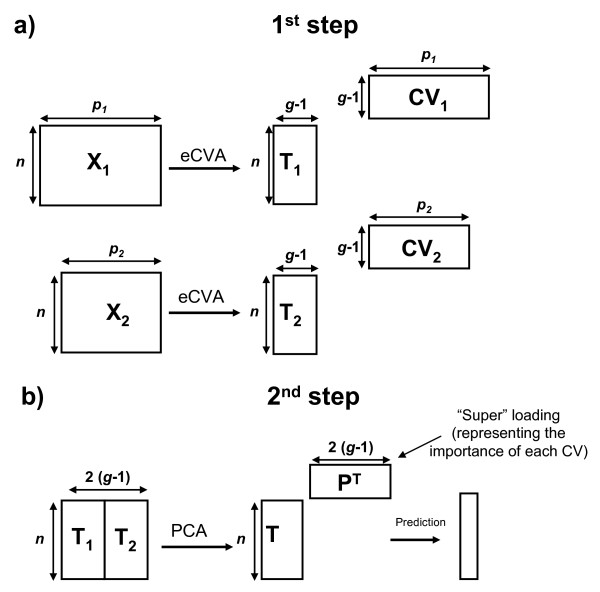
**Architecture of the mid-level fusion analysis employed here on two data sets X_1_and X_2_**. The same *n *samples are divided in *g *groups. a) eCVA is applied on each data set to determine the Canonical Variates **CV_1_**and **CV_2_**allowing the best discrimination and the corresponding scores **T_1_**and **T_2_**. b) The scores are merged and analyzed using PCA. The global scores **T **and super loadings **P^T^**are obtained. Class prediction is obtained based on **T**.

The purpose of data fusion is to extract the information spread across the different platforms. Conclusions based on multiple independent types of measurement are more reliable than from one platform alone (assuming that each type of measurement is relevant to the investigated problem). More importantly it may provide new scientific insights on relations between compounds such as protein-metabolite interactions. The simplest approach is to concatenate the different data sets in a low-level fusion approach [16]. However this strategy is greatly affected by the variability between the two platforms. Therefore we designed a mid-level architecture capable to discriminate between a number of beforehand defined groups. The mid-level fusion analysis contains two steps as presented in Figure 1.

Two problems have to be faced during the analysis of the two -omics data set. The first one has to deal with a dimensionality problem. Indeed both metabolomics and proteomics studies measured a large number of variables on relatively few samples. The second challenge is related to the missing values. Each instrumental platform received the same samples. However, some measurements had to be excluded from the study because of instrumental artifacts or technical issues during the measurement. Therefore the algorithm has to deal with two different problems. In the two following sections we provide answers for each challenge. Finally, we discuss how to integrate these two steps and how to interpret the final results.

#### First step: extended Canonical Variate analysis

The first step consists of extracting and compressing the information contained in each data set. PCA [27] is the most usual way to compress data [28]. However, biological data are often affected by multiple sources of variance. Therefore, we opted for a "LDA-like" (Linear Discriminant Analysis) method. The advantage of this approach is to focus only on the information related to the defined classes (here the different groups of rats). Yet such methods are confronted with the "curse of dimensionality". Indeed LDA requires more samples than the variables, otherwise the inversion of the within-group covariance matrix becomes impossible. A recently proposed algorithm provides a solution. Extended Canonical Variate Analysis (eCVA) [17,29] has been developed to cope with multi-collinear data. The concept of this method very much resembles Fisher LDA. Assuming a data matrix **X **(*n *× *p*) where *g *groups of *n_i _*samples are present, the within-group covariance matrix **S_within _**is defined as:(1)

and the between-group **S_between _**is:(2)

where ***x_ij _***is the *j*th sample in the *i*th group,  is the mean vector in the *i*th group, and  is the overall mean vector. The best discrimination between the groups is obtained by defining a direction ***w ***maximizing the ratio of the between-group on thewithin-group covariances [28]:(3)

When **S_within _**is non-singular, the equation (3) can be rewritten in the form of an eigenvalue problem:(4)

where *a *represents the number of directions, *λ_a _*are the eigenvalues and **w**_a _the eigenvectors. Equation (4) has a maximum dimensionality of *a *= min(*p*,*g*-1). Therefore even in the case of high dimensional data, the number of canonical variables extracted is equal to the number of groups minus one (*g-*1). It can be shown that this problem (equation (4)) can be turned into a regression problem [17]. Partial Least Squares (PLS) method is then used to solve the equation (5) corresponding to this regression problem:(5)

where **Y **contains the differences , the columns of **B **are **w**_a _and **F **is a residual matrix. The scores **T **can then be calculated projecting them along the directions **w**_a _i.e. the canonical variates (**CV**). The classification model is then constructed using these scores. Note that the best results where obtained on vast scaled data [30] (compared to autoscaled, Pareto scaled and mean centered data).

The two data sets here have very different dimensions. The proteomics data set contains almost 50 times more variables than the metabolomics data. Therefore an additional variable selection step was introduced for the proteomics platform. The eCVa model was constructed on 7114 variables. The 153 most important variables were selected and used to reconstruct the eCVA model. The latter is then used in the rest on the analysis.

#### Second step: Principal Component Analysis for missing values

The information related to groups has been compressed by eCVA into a small set of (*g*-1) canonical variates. Therefore, the problem of dimensionality does not apply anymore in the second step. The experimental design is such that the same samples should be measured by all platforms. In practice some samples were either missing or the obtained measurements were detected as outliers during the exploratory analysis. As a consequence, some data points are missing in the concatenation of **T_1 _**and **T_2_**. It is important to note that this problem does not arise in the previous step because the different eCVA models are constructed per platform. Therefore, if a sample is missing it is simply excluded from the construction of the eCVA model. One should note that the quantity of missing data should not unbalance dramatically the number of samples per group (this was not observed in the described data). However, if this strategy is applied in the second step a considerable amount of information would remain unused (the samples measured by one platform but not the other). A method able to deal with missing values must therefore be used. We propose an adaptation of the PCA based on the missing toolbox from Claus Andersson [31,32]. The missing elements are replaced by model estimates of these elements. The estimates are iteratively improved until a convergence criterion is fulfilled and the estimates do not change significantly. The influence of the missing values on the model is then limited [33,34].

#### Validation - Interpretation

The method proposed here explicitly uses class information. A risk of overfitting exists; therefore a validation procedure has to be implemented. A test set is constructed using 20% of the samples. The test samples must be representative of the whole set. They are selected using Kennard and Stone algorithm [35]. The test samples are then inputted in the eCVA model and then projected in the PC space. The prediction can be obtained using an approach similar to Principal Component Discriminant Analysis. Once validated, the model is reconstructed using all available samples.

The biological interpretation is performed by assessing the importance of the original variables. It is calculated by multiplying the canonical variates from the first step by the loadings of the PCA (second step). For example the weights of the original variables from the first platform are calculated as follow:(6)

Where **w_1 _**contains the weights of the original variables for the global model (*i.e*. loadings) **P_:,1:g-1 _**contains the importance of the *g*-1 first latent variables. The same operation can be done for the second platform using the corresponding *g*-1 latent variables. The model can be inspected using a score plot and/or biplot.

The most important variables are then put into context by projecting them into a correlation network. This network is constructed by calculating all pair-wise Pearson correlations  in the different classes:(7)

Where *n_i _*is the number of samples,  and  are the sample means of *x*_1 _and *x*_2 _while and are the standard deviations of *x*_1 _and *x*_2_. The complete network is too large and complex to be used. Therefore a sub-network is represented using the most important variable as a seed which is expanded to all variables with a correlation superior to 0.8 (in absolute value) to them. The network is visualized using Graphviz [36,37] where each node represents one variable. The node is a square if it represents a variable seen as important by the fusion method, otherwise as an ellipse.

## Results

### Explorative analysis

**Figure 2:**
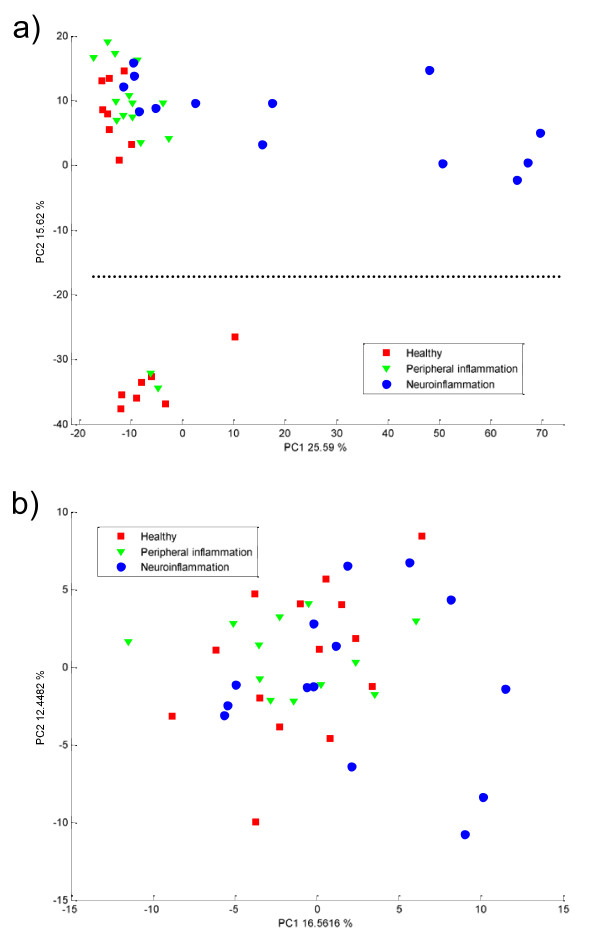
**PCA score plot obtained on a) proteomics data and b) metabolomics data after autoscaling**. The healthy and inflammation controls are represented as red squares and green triangles. The disease samples are in blue circles. The dotted line in a) represent the separation between the two batches of measurements.

The analysis of the data can be performed first per platform. This is particularly relevant during the exploratory analysis. PCA was used to visualize each dataset and detect eventual outliers. In Figure 2 score plots obtained for both platforms are presented. The samples are colored in accordance to their group memberships. One can observe that the separation is clearer for the proteomic platform than for the metabolomics. Indeed in Figure 2a the main source of variance (PC1 explaining 25.5% of the variance) allows one to separate most of the disease group (blue circles) from the control samples (in red for healthy and in green for inflamed animals). Interestingly, the PC2 also shows separation of two groups of points. This source of variation represents 15% of the variance. However, it does not correspond to the experimental design. This separation is then certainly due to either biological or instrumental variations. In this case the separations in two subgroups correspond to two series of measurements. This effect could be corrected for with dedicated method [38]. However the proposed fusion method should be generic enough to deal with this issue. Therefore we did not investigate further batch effects. The equivalent plot for the metabolomics platform is presented in Figure 2b. Here no instrumental variations are observed but the disease group is also strongly overlapping with the control samples.

**Figure 3:**
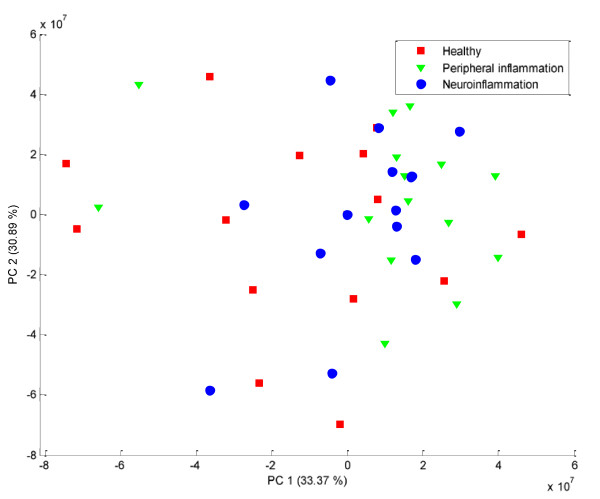
**Score plot obtained on the concatenation of the proteomics and metabolomics data sets after autoscaling and PCA**. The healthy and inflammation controls are represented in red and green. The disease samples are in blue. The three classes overlap completely.

The most straightforward approach for data fusion is to analyze the two data sets together. This is called low-level fusion [16]. It is expected that the two types of information should complete each other and improve the class separation. This low level fusion provides disappointing results, as can be seen in Figure 3. The two data sets are analyzed together, using PCA for missing values. The three classes are overlapping in the plane defined by PC1 and PC2 (explaining 64% of the variance).

Both instrumental analyses provide different information about the group separation. However, in both cases the biological or instrumental variations distort the picture. The explorative analysis of both sets as one unique data matrix does not provide a better but actually a worse separation of the groups. In that respect it is relevant to use a supervised method in order to extract the relevant information from each data set. This can be performed using mid-level fusion as described in the methods section.

### Mid-level fusion

Our approach is based on two steps. The first one consists of a supervised analysis of each platform. After the first step, each submodel (*i.e*. per platform) can be assessed in terms of complexity and prediction, based on a cross validation included in eCVA. Here the inner PLS loop of the eCVA models for the proteomics and metabolomics platforms are using respectively 4 and 8 latent variables. As additional check, we perform the prediction of the test samples left out during the construction of the eCVA models. They were correctly predicted in 78% of the cases by the proteomic platform and in 78% by the metabolomics platform. The latent variables constructed by eCVA are inspected in order to determine whether the variability spanned by the latent variables from one model is comparable to the one spanned by the other model. The latent variables obtained for each platform are then concatenated. The newly formed matrix can be analyzed using PCA. The test samples groups can be predicted using a method similar to Principal Component Discriminant Analysis (PCDA) [39]. The fusion leads to 89% of correct classification on the validation set.

**Figure 4:**
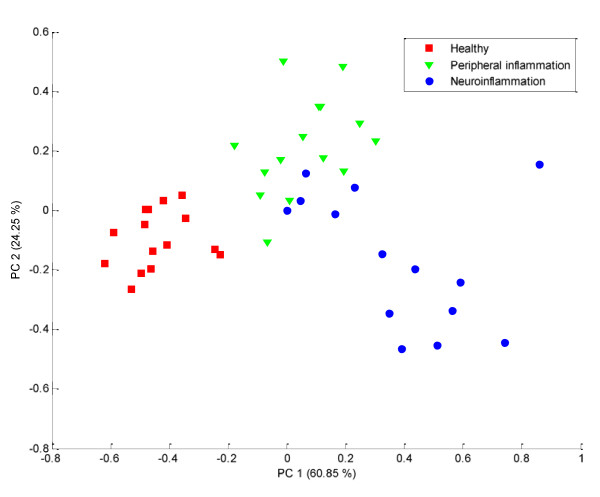
**PCA score plot allowing the visualization of the results of the fusion of proteomics and metabolomics platforms**. The samples are color-coded according to group labels: in red squares the healthy control, in green triangles the inflammation group and in blue circles the disease group.

Since the model shows good predictive ability we consider it as statistically validated. The model is reconstructed using all available samples before starting interpretation. The resulting model can be graphically assessed using a score plot as shown in Figure 4 where each sample is color coded according to the groups' label. The separation of the healthy control (in red) from the two other groups can be seen along PC1. The disease and inflammation groups are mostly separated from each other along PC2 with some overlap. It is interesting to note that the spread of the disease group is larger than the other groups. It suggests that this group is more heterogeneous. Part of the neuroinflammation group is overlapping with the peripheral inflammation group. This would suggest that the neurological symptoms are delayed for the corresponding animals.

The most significant metabolites and proteins linked to the disease group are then studied. The selection rules for proteins were that at least three peptides must show similar behavior (for example up or down regulation in the disease group). Their importance is then averaged. The importance is a relative measure of the influence of the variable on the definition of the group considered (*i.e*. the projection of the loading on the vector defined by the mean of the group). In Table 2 a selection of the names of main variables, the corresponding Uniprot/CAS numbers and their relative importance to define this group is listed. The sign of these values indicates whether the metabolite/protein concentration is up or down regulated in the disease group in comparison to the controls. The values have been normalized in such way that the most important variable (Hemopexin) has a value of 1.

**Table 2:** Selection of metabolites and proteins up or down regulated in the disease group.

Name	UniProt/CAS registration number	Importance to define disease group
Hemopexin	P20059	1.00

δ 2.22633		-0.82

T-kininogen 1	P01048	0.80

Serum albumin	P02770	-0.68

T-kininogen 2	P08932	0.67

Complement C3	P01026	0.67

Serotransferrin	P12346	0.66

Haptoglobin	P06866	0.58

Ceruloplasmin	P13635	0.50

Acetone	67-64-1	-0.49

Succinate	110-15-6	-0.46

δ 3.63769		-0.34

Glyceric acid	473-81-4	0.33

Glutamine	56-85-9	-0.32

Valine	72-18-4	-0.32

Sucrose	57-50-1	-0.29

Glyceric acid	473-81-4	0.29

Complement C4	P08649	0.29

δ 2.24436		-0.28

δ 2.32502		-0.26

Citrate	77-92-9	0.25

δ 4.0078		0.25

δ 4.01128		0.24

3-hydroxybutyrate	625-72-9	-0.23

Lysine	56-87-1	0.23

Pantothenate	79-83-4	-0.22

Unidentified signals are reported using chemical shift (δ).

**Figure 5:**
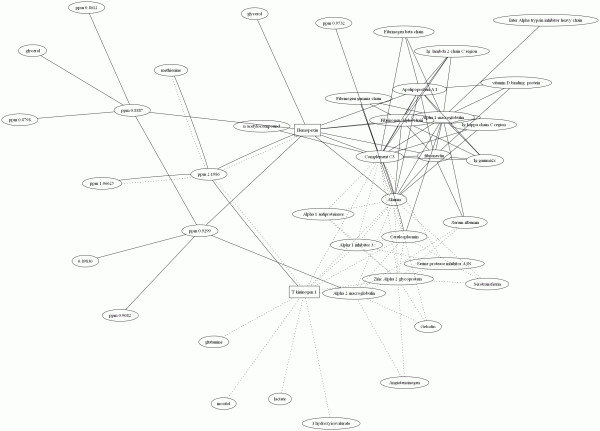
**Correlation network centered on hemopexin (P20059) and T-kininogen 1 (P01048)**. The two first layers of correlation are represented. The correlations observed in the healthy control samples are represented by solid black line, the ones in the disease group by dotted lines.

The interpretation can obviously be based on an univariate approach *i.e*. considering each selected variable separately. However, the interest of fusion is to make a connection between the two types of information and to evaluate the link between proteins and metabolites. To facilitate the interpretation of the results we constructed a correlation network that embodies the interconnections between the protein and metabolite variations. The result is presented in Figure 5. Each node corresponds to one metabolite or one protein. The links between nodes indicate that a correlation is superior to 0.8 (or inferior to -0.8). The complete network is too complex to be reproduced in a single image. The Figure 5 represents a portion of the complete correlation network centered on hemopexin and T-kininogen 1 *i.e*. the variable seen as most important (see Table 2). Only the proteins and metabolites directly correlated to hemopexin or T-kininogen 1 or through one other variable are represented. The correlation network is clearly different between the disease and control groups. The correlations observed in the healthy control are represented by solid black line. The correlations observed in the disease group are represented by dotted lines.

## Discussion

A fingerprint of the EAE onset is defined by our data analysis approach. The first aspect to be discussed is the statistical performance of our method. The obtained results show better prediction ability than individual analysis and low level fusion based on either eCVA or Partial Least Squares Discriminant Analysis (PLS-DA) (see the additional file 1). The limitations of this approach are mostly related to the statistical aspects. Firstly, the underlying concept of both eCVA and PCA is that the information is related to linear variations. In the cases of more complex signals (non linear responses), the obtained discrimination could be too low or based only on a part of the metabolites and proteins really involved. Secondly, the number of samples measured by both platforms should be sufficiently high to ensure that the relations observed are not artifacts.

The second aspect to be discussed is the nature of the variables selected. One can glean from Table 2 that both proteins and metabolites participate in the discrimination model. Apparently the fingerprint of the EAE onset is better defined using both proteomics and metabolomics knowledge *i.e*. using a systems biology approach.

In terms of biological interpretation, the obtained results are coherent with previous studies. Indeed the proteins presented in Table 2 have been previously linked to EAE and/or MScl. Increased levels of hemopexin and T-kininogen 1 have been previously reported in neurological disorders [40-42]. The expression level of the kininogen 1 receptor has been proposed as a non-specific index of disease activity in MScl by Prat et al. [43]. Serum albumin [41,42] is known to cross the blood brain barrier after administration of CFA and, in absence of inflammatory cells in the CNS, albumin is not triggering any neuroinflammation [44]. However, we observed reduced level of albumin in the disease group. This suggests a relation between the neuroinflammation process and the serum albumin present in the CNS. However this observation needs to be validated. Complement C3 [45-48] and Complement C4 [45,49] have been related to MScl. In addition the Complement C3 and its derived fragments have been marked as a central player in the pathophysiology of EAE [50]. Increased concentration of Haptoglobin has been found in MScl [45]. It has been suggested that Haptoglobin plays a modulatory and protective role on autoimmune inflammation of the CNS [51]. Ceruloplasmin [41,45,52] and citrate [53] are known to rise in concentration during the inflammation phase. Similarly some of the selected metabolites have been ascribed to MScl. Increased lysine level is known to have an impact on the entry of arginine in leucocytes and thus on the NO synthesis [54]. The complete biological interpretation of this multivariate model requires larger discussion and validation. These aspects are not in scope of this manuscript and will not be discussed for brevity reasons.

Figure 5 gives a more global view of the problem. First, one finds that the correlation network is changed by the disease. Therefore we decided to focus on two of the selected variables (Hemopexin and T-kininogen 1) and the variables directly correlated to them. Here one can observe that multiple correlations to T kininogen 1 appear in the disease group. This would indicate that the normal pathways are changed and a new pathway involving T kininogen 1 as central player and a number of metabolites (e.g. lactate or inositol) and proteins (e.g. Complement C3 or Ceruloplasmin) is either created or becoming preeminent.

## Conclusions

The feasibility of fusion of multiple omics platforms was discussed in this work. The study of the onset of EAE was chosen as a case study. From the point of view of data analysis multiple challenges had to be addressed. The first one had to do with the biological variation usually encountered in omics experiments. The method proposed here focuses on the information of interest through a training procedure. However, the high dimensionality of the data is problematic for discriminant methods. This explains why it is necessary to use eCVA, since this method is able to handle highly collinear multivariate data. The second difficulty is linked to the multiplatform aspect of this work: the same samples have been measured by two platforms. In the process some measurements were either lost or discarded for multiples reasons (missing samples, instrumental problems, contamination, and outliers). We propose to apply in the last step of the fusion a version of PCA which is able to cope with missing values.

Overall the results of the fusion show a significantly better discrimination of the three classes (disease, healthy control and inflammation control) compared to individual analysis of the two data sets. A second advantage here is the possibility of studying the correlation between proteomics and metabolomics without having to assume any model (such as the ones based on known pathways). For that purpose, we propose the use of correlation networks. The variables seen as important for any discriminant model can be put in context using this approach. The obtained networks give an insight about the modification of the metabolic pathways by the disease.

From a biological point of view the selected variables appears to make sense. The proteins and metabolites described in this study were previously found in relationship to the EAE and Mscl and therefore provide a biological validation for the fusion of data from two different platforms. Performing a fusion of several platforms can further confirm the role of the described pathways and potentially highlight new pathways involved in the EAE disease process. Future work will focus on the search and interpretation of newly detected metabolites and proteins. The pattern defined by these variables must also be studied by itself and put into the context of system biology.

## List of abbreviations

CFA: Complete Freund Adjuvant; CNS: Central Nervous System; CSF: Cerebrospinal Fluid; eCVA: extended Canonical Variates Analysis; LDA: Linear Discriminant analysis; MScl: Multiple Sclerosis; MBP: Myelin Based Protein; NMR: Nuclear Magnetic Resonance; PCA: Principal Component Analysis.

## Competing interests

The authors declare that they have no competing interests.

## Authors' contributions

This study was designed by AA, TT, TL, SSW, and LMCB. The protocol related to the EAE experiment was designed by AA and TT. The animal experiments were carried out by AA, HvA and ES. The NMR measurement protocol was designed by AS, KA and SSW. The sample preparation, NMR measurements and data processing were made by AS. The Orbitrap measurement protocol was designed by MS and TL. The sample preparations, Orbitrap measurements, and data preprocessing were made by MS. The fusion method presented here was developed and applied by LB. The redaction of this manuscript was done by LB, AS and MS. LMCB, AA, SSW and TL have been involved in the critical revision of the manuscript. All authors read and approved the final manuscript.

## Supplementary Material

Additional file 1 **Supplementary Material**. The low level fusion has been investigated and is presented in the additional file 1 together with the effect of block scaling. The comparison between results of eCVA and Partial Least Squares - Discriminant Analysis is also provided in this document. Click here for file
