# Supplementary material for: Fusion of metabolomics and proteomics data for biomarkers discovery: case study on the experimental autoimmune encephalomyelitis
Source: BMC Bioinformatics. 2011 Jun 22;12:254. doi: 10.1186/1471-2105-12-254 (PMC3225201; doi:10.1186/1471-2105-12-254)
Supplement: Additional file 1 — Supplementary Material. The low level fusion has been investigated and is presented in the additional file 1 together with the effect of block scaling. The comparison between results of eCVA and Partial Least Squares - Discriminant Analysis is also provided in this document. [file 1471-2105-12-254-S1.DOC]

# Fusion of metabolomics and proteomics data for biomarkers discovery: case study on the experimental autoimmune encephalomyelitis

# Supplementary materials

Lionel Blanchet^1^, Agnieszka Smolinska^1^, Amos Attali^2^, Marcel P. Stoop^3^, Kirsten A.M. Ampt^1^, Hans van Aken^2^, Ernst Suidgeest^2^, Tinka Tuinstra^2^, Sybren S. Wijmenga^1^, Theo Luider^3^ and Lutgarde M.C. Buydens^1^

^1^ Institute for Molecules and Materials, Radboud University Nijmegen,

Nijmegen, The Netherlands

^2^ Abbott Healthcare Pharmaceuticals Nederland B.V.

Weesp, The Netherlands

^3^ Department of Neurology, Erasmus University Medical Center

Rotterdam, The Netherlands

**Results obtained on separate platforms**

The supervised data analysis per platform was performed using extended Canonical Variates Analysis (eCVA). As explained in methods section, the principle of this method is very similar to Fisher Linear Discriminant Analysis. The main breakthrough of this method is to be able to deal with more variables than samples. In such situation another method is usually applied: Partial Least Squares Discriminant Analysis (PLS-DA). Table S1 present here the best prediction results obtained by both methods. The application of PLS-DA was done using the implementation proposed by Gidskehaug *et al*.[1] including a cross model validation. This algorithm is therefore providing a list of significant variables used by the regression models.

Table S1: Predictions obtained by eCVA and PLS-DA during the individual analysis of the two platforms.

| Method | Platform | Nb LVs | Nb variables | Prediction |
| --- | --- | --- | --- | --- |
| ECVA | Proteomics | 4 | - | 78% |
| ECVA | Metabolomics | 8 | - | 78% |
| PLS-DA | Metabolomics | 1 | 153 | 50% |
| PLS-DA | Proteomics | 2 | 500 | 63% |

The eCVA outperforms the PLS-DA results in both cases. The number of latent variables used is more important for eCVA. However one should remember that this corresponds only to the inner PLS loop of the algorithm and therefore doesn’t represent directly the complexity of the eCVA model.

**Results obtained using a low level fusion approach**

The simplest form of data fusion consists in concatenating the two data sets together and performing then data analysis. Such approach has been applied to the joint analysis of our proteomics and metabolomics data sets. The analysis was based on two supervised methods. The first one is the eCVA, also used in the mid level fusion architecture proposed in the main text. The second one is the well known PLS-DA.

Some inconvenient arise when using low level fusion. First the problem of missing values must be dealt with immediately. Here we applied the strategy of complete cases [2]. Secondly, the two data sets differ largely in term of size and amount of variation and structure. Therefore the effect of blockscaling is expected to be crucial in low level fusion. We investigate the results with and without block scaling. The block scaling used here equalizes the variation of both data matrices [3].

The Table S2 regroups the obtained prediction on an independent test set. The numbers of latent variables (LVs) of the PLS-DA models and inner PLS loop for eCVA are also provided.

Table S2: Parameters used and predictions obtained by eCVA and PLS-DA in a low level fusion.

| Method | Block Scaling | Nb LVs | Nb variables | Prediction |
| --- | --- | --- | --- | --- |
| ECVA | no | 5 | - | 50% |
| ECVA | yes | 23 | - | 50% |
| PLS-DA | no | 2 | 400 | 40% |
| PLS-DA | yes | 2 | 150 | 60% |

One could expect the low level fusion to perform at least as good as the worst individual analysis [4]. Nevertheless this is not observed for eCVA. This is maybe due to the effect of missing values. In any cases the models obtained have no predictive power. Therefore no biological conclusions can be drawn from these models. It appears that the differences in variability and in data structure forbid the use of low level fusion.

**References**

1. Gidskehaug L, Anderssen E, Alsberg BK: **Cross model validation and optimisation of bilinear regression models** *Chemom Intell Lab Syst* 2008, **93**(1):1-10.

2. Arteaga F, Ferrer-Riquelme AF: **Missing data**. In: *Comprehensive chemometrics.* Edited by Brown SD, Tauler R, Walczak B, vol. 3. Amsterdam: Elsevier; 2009: 285-314.

3. van den Berg RA, Rubingh CM, Westerhuis JA, van der Werf M, Smilde AK: **Metabolomics data exploration guided by prior knowledge**. *Anal Chim Acta* 2009, **651**(2):173-181.

4. Forshed J, Idborg H, Jacobsson S, P.: **Evaluation of different techniques for data fusion of LC/MS and ^1^H-NMR**. *Chemom Intell Lab Syst* 2007, **85**(1):102-109.
